# The process of developing a score to measure dementia‐friendliness in African American faith communities

**DOI:** 10.1002/alz70861_108888

**Published:** 2025-12-23

**Authors:** Alexis A Bender, Grayson Baxter, Nidhi Ramprasad, Fayron Epps, Miranda A Moore

**Affiliations:** ^1^ Emory University, Atlanta, GA USA; ^2^ UT Health San Antonio, San Antonio, TX USA

## Abstract

**Background:**

As the prevalence of dementia rises, organizations and communities have recognized the importance of having dementia‐friendly spaces and are developing programs to meet the needs of people living with memory loss and their families. Despite the increase in dementia‐friendly programs and settings, there is a dearth of tools to assess and measure the level of dementia‐friendliness in small communities such as churches and community centers.

**Method:**

In partnership with African American church communities across the state of Georgia, we developed a person‐centered Dementia‐Friendly Community Assessment tool in multiple phases. Following our initial pilot testing and refinement through focus groups with church members, leaders, and advisory board members, we finalized and fielded a survey across 15 churches. The survey included questions about resources and environment modifications; dementia knowledge and skills; and three separate dementia‐related stigma scales administered based on self‐identified role. Following univariate and bivariate analyses, we conducted principal component analysis with oblique rotation to reduce items and identify the factor structure. We then conducted confirmatory factor analysis and assessed each component and the overall scale for reliability. Analyses were conducted using Stata v.18.5 and MPlus v.8.11.

**Result:**

1,162 participants completed the survey. Nearly half (48.9%) of the participants were 60 or older and had at least a bachelor’s degree (48.45%). Just under 10% (9.3%) self‐identified as living with memory loss while 184 participants (15.8%) said they knew someone with memory loss and 694 people (59.7%) provided care to someone living with memory loss. Final analysis confirmed a five‐component scale: Information, Resources, Access, Worship, and Resource saturation with excellent model fit. Each individual component had good reliability (Cronbach’s alpha range 0.76 – 0.84) and overall scale reliability (α = 0.88). Higher overall scores were significantly associated with better scores on dementia knowledge and skills, and lower stigma on all stigma scales.

**Conclusion:**

This tool demonstrates appropriate and reliable measurement of dementia friendliness in relatively homogenous church communities and accommodates diverse geographic locations (i.e., urban, rural, suburban) and community sizes. The five components can be scored individually to assess areas of strength or improvement or used as an overall measure of dementia‐friendliness.

